# Integrated Clinical Safety Assessment of Orally Administered Antimicrobials from Different Pharmacological Classes in Nile Tilapia (*Oreochromis niloticus*)

**DOI:** 10.3390/ani16142188

**Published:** 2026-07-14

**Authors:** Camila Carlino-Costa, Susana Luporini de Oliveira, Mayumi Fernanda Aracati, Letícia Franchin Rodrigues, Hélio José Montassier, Camila Ferraz Franciscão, Maria Eduarda Pultz, Karine de Sousa Araujo, Ives Charlie-Silva, Luiz Arthur Malta Pereira, Marco Antonio de Andrade Belo

**Affiliations:** 1 Department of Pathology, Reproduction and One Health, Sao Paulo State University (UNESP), Jaboticabal 14884-900, SP, Brazil; camila.carlino@unesp.br (C.C.-C.); susana.luporini@unesp.br (S.L.d.O.); mayumiaracati@hotmail.com (M.F.A.); leticia.franchin@unesp.br (L.F.R.); helio.montassier@unesp.br (H.J.M.); 2 Laboratory of Animal Pharmacology and Toxicology, Brazil University, Descalvado 13690-000, SP, Brazil; camila.f.franciscao@gmail.com (C.F.F.); mariapultz11@gmail.com (M.E.P.); karine.saaraujo@gmail.com (K.d.S.A.); charliesilva4@gmail.com (I.C.-S.); luiz.pereira@ub.edu.br (L.A.M.P.)

**Keywords:** fish pharmacology and toxicology, aquaculture, β-lactams, macrolides, sulfonamide–diaminopyrimidine combinations

## Abstract

The rational use of antimicrobials in aquaculture requires rigorous clinical safety assessments to ensure fish health and to minimize toxicological risks associated with drug administration. This study evaluated the clinical safety of orally administered antimicrobials incorporated into feed, representing different pharmacological classes, in Nile tilapia (*Oreochromis niloticus*). Three bioassays were conducted for sulfadimethoxine combined with ormetoprim (SDM–OMP, 50, 100, and 200 mg·kg^−1^, at a fixed 5:1 SDM:OMP ratio), spiramycin (SPM, 40, 80, and 160 mg·kg^−1^), and phenoxymethylpenicillin potassium (PMP, 10, 20, and 40 mg·kg^−1^), including control and treated groups at different dose levels, followed by a drug-free recovery period. The assessment integrated hematological parameters, serum biochemical analyses, and histopathological evaluations of the liver, kidney, and spleen. Exposure to the antimicrobials resulted only in mild, transient, and reversible physiological alterations, with no evidence of clinically relevant toxicity or structural lesions in the analyzed organs. Collectively, these findings demonstrate that SDM–OMP, SPM, and PMP exhibit a favorable clinical safety profile in Nile tilapia, even at doses up to four times the recommended therapeutic level, supporting their responsible use in aquaculture under controlled conditions.

## Simple Summary

The use of antimicrobial drugs is common in aquaculture for the control of bacterial diseases; however, ensuring the safety of these products for fish health is essential. When improperly used, antimicrobials may cause undesirable effects in organisms and compromise the sustainability of aquaculture production. In this study, we evaluated the safety of the oral administration of three commonly used antimicrobials incorporated into the feed of Nile tilapia. Fish were exposed to the drugs for a defined treatment period, followed by a drug-free recovery phase. Hematological parameters, liver and kidney function, as well as the histological structure of internal organs, were assessed. The results revealed only mild and transient alterations, with no clinically relevant damage or permanent lesions. After the recovery period, all evaluated parameters returned to baseline levels. These findings indicate that the antimicrobials assessed are safe for Nile tilapia under the tested conditions and support the responsible use of antimicrobial drugs in aquaculture.

## 1. Introduction

Aquaculture currently accounts for more than 50% of global aquatic food production, establishing itself as one of the pillars of global food security [1]. In this context, global aquaculture production reached 130.9 million tons in 2022, reflecting the increasing intensification of production systems [1]. Among cultivated freshwater fish species, Nile tilapia (*Oreochromis niloticus*) occupies a prominent position in global production, with Brazil ranking among the world’s leading producers and reaching one million tons in 2025 [2].

The intensification of aquaculture production, although essential to meet the growing demand for food, has been accompanied by significant sanitary challenges, including increased stocking density, chronic stress, and deterioration of water quality, all of which favor the occurrence of infectious diseases [3]. Among these, bacterial diseases represent the main cause of outbreaks in aquaculture systems, accounting for approximately 55% of reported cases, with direct impacts on mortality, animal welfare, and economic losses estimated in billions of dollars annually [4,5].

Although preventive strategies such as good management practices, biosecurity measures, adequate nutrition, and environmental monitoring are fundamental to reducing disease incidence, bacterial outbreaks may still occur in production settings and require targeted therapeutic interventions [6,7]. In such contexts, the use of antimicrobials may represent an important tool for infection control and mitigation of production losses, provided that their use is guided by robust scientific evidence and grounded in the principles of rational use, taking into account animal safety, environmental impacts, and the risk of selecting antimicrobial-resistant bacteria [8]. The clinical safety evaluation of antimicrobial use in aquaculture is essential for both production sustainability and food safety [6]. Because antimicrobials are mainly administered through medicated feed, understanding their physiological effects is necessary to establish safe treatment protocols and support rational use while minimizing the risks of drug residues in edible tissues and the emergence of antimicrobial resistance [9].

In Nile tilapia farming, antimicrobial therapy is commonly employed to control bacterial diseases such as streptococcosis (caused mainly by *Streptococcus agalactiae* and *S. iniae*), motile aeromonad septicemia (*Aeromonas hydrophila*), columnaris disease (*Flavobacterium columnare*), and infections associated with *Edwardsiella* spp [10]. Antimicrobial classes commonly used in aquaculture include quinolones, tetracyclines, phenicols, and sulfonamides, depending on regional regulations and pathogen susceptibility profiles [11,12]. Among antimicrobials of relevance to veterinary medicine and aquaculture, representatives of sulfonamide–diaminopyrimidine combinations, macrolides, and β-lactams stand out, as these classes are widely recognized and included in the World Organization for Animal Health List of Antimicrobials of Veterinary Importance [13]. The selection of drugs from these classes allows the evaluation of contrasting pharmacological profiles involving different mechanisms of action, pharmacokinetic characteristics, and potential systemic effects. Sulfadimethoxine combined with ormetoprim (SDM-OMP) represents a synergistic antimicrobial combination that inhibits sequential steps of bacterial folate synthesis [14]. This association has been historically investigated in fish due to its relatively favorable oral bioavailability and wide tissue distribution [14]. In addition, SDM-OMP undergoes extensive hepatic metabolism and may interfere with hematological parameters, particularly under prolonged exposure or high doses, highlighting the importance of evaluating its systemic safety in aquaculture species [15,16]. Spiramycin, a macrolide antibiotic, is characterized by extensive intracellular penetration and immunomodulatory properties that may influence host inflammatory responses during infection. Macrolides can accumulate in immune cells and modulate cytokine production and inflammatory pathways, suggesting potential effects on immune and inflammatory responses that are relevant when evaluating systemic clinical safety [17,18]. Phenoxymethylpenicillin potassium (PMP) is a β-lactam antibiotic with a pharmacokinetic profile favorable for oral administration [19]. Although many bacterial pathogens affecting fish are Gram-negative, several major diseases in Nile tilapia aquaculture are caused by Gram-positive bacteria, including *Streptococcus agalactiae*, *Streptococcus iniae*, and *Lactococcus garvieae*, which are responsible for outbreaks associated with significant economic losses [20,21]. These pathogens are generally susceptible to β-lactam antibiotics that inhibit bacterial cell wall synthesis. However, β-lactams such as PMP may exhibit limited stability in aquatic environments [22,23], which can potentially reduce their therapeutic effectiveness when drugs are directly dispersed in water. The use of medicated feed, however, allows controlled oral delivery and may partially overcome this limitation by ensuring ingestion of the compound by the fish. Despite extensive information available for β-lactams in mammals, data regarding their physiological effects and clinical safety in teleost species remain limited, particularly following oral administration via medicated feed.

Pharmacokinetic studies of antimicrobial agents in fish have shown that drug absorption, distribution, metabolism, and elimination may differ substantially from those observed in terrestrial animals due to species-specific physiology, environmental temperature, and aquatic exposure routes [24,25]. For example, variable elimination half-lives, tissue distribution patterns, and metabolic pathways have been reported for sulfonamides, tetracyclines, and macrolides in teleost fish [26]. Consistent with these findings, evidence of hematological and oxidative responses in tilapia exposed to sulfonamides has been reported under controlled administration regimens [27,28], and macrolide exposure (e.g., azithromycin) has been evaluated through biomarker-based approaches in tilapia [29]. In parallel, the interpretation of hematological and biochemical alterations in fish requires caution because these endpoints are influenced by physiological and environmental variability, which can reflect adaptive responses rather than clinically relevant toxicity when not supported by convergent evidence from multiple systems [30,31,32,33]. Therefore, experimental designs that integrate hematology, serum biochemistry, and tissue-level evaluations are particularly valuable to distinguish transient physiological adjustments from systemic toxicological effects [30,31,32,33].

To our knowledge, no previous study has comparatively evaluated the clinical safety of SDM-OMP (50, 100, and 200 mg·kg^−1^, at a fixed 5:1 SDM:OMP ratio), SPM (40, 80, and 160 mg·kg^−1^), and PMP (10, 20, and 40 mg·kg^−1^) administered orally via medicated feed to Nile tilapia under a standardized experimental design. Therefore, the objective of this study was to assess the systemic clinical safety of these antimicrobial agents using a controlled multi-dose exposure protocol including treatment and drug-free recovery phases. Physiological responses to drug exposure were investigated through the integration of hematological parameters, serum biochemical markers, and histopathological evaluation of key organs (liver, kidney, and spleen), providing comparative baseline evidence on systemic tolerance and potential toxicity associated with these antimicrobial classes in intensive aquaculture systems.

## 2. Materials and Methods

### 2.1. Fish

For the clinical safety study, three experimental trials were conducted simultaneously to evaluate the SDM-OMP combination (Trissulfin^®^ SID, Ourofino, Cravinhos, Brazil), PMP (Pen-Ve-Oral^®^, Eurofarma, Itapevi, Brazil)and SPM (Rovamycine^®^, Sanofi, Susano, Brazil). For each antimicrobial, 128 Nile tilapia juveniles (mean weight: 80 g; mean length: 15 cm; age: ~60 days) were used, totaling 384 animals, all originating from the same spawning batch (Piscicultura Projeto Peixes, Sales Oliveira, SP, Brazil—20.93282 South latitude and 47.83857 West longitude, in the state of São Paulo). Fish were randomly allocated by body weight into 48 tanks (130 L each, *n* = 8 per tank, corresponding to an approximate biomass density of 4.92 kg m^−3^) supplied with running water devoid of chlorine, coming from an artesian well with a flow rate of 1 L/min. After transport to the experimental facilities of the Animal Pharmacology Laboratory (Brazil University, Descalvado, SP, Brazil), fish were acclimated for 15 days, a period required for plasma cortisol concentration and osmolarity to return to baseline levels. During the first three days of acclimation, animals were subjected to salt baths using a NaCl solution at a concentration of 6.0 g·L^−1^ [34]. Fish were fed a commercial extruded diet as the basal feed twice daily, corresponding to 2% of the tank biomass according to the nutrition balance described in item 2.4. Water quality parameters were monitored twice daily throughout the experimental period using a YSI-63 pH meter and a YSI-55 dissolved oxygen meter, and remained within the ranges considered adequate for the welfare of tropical fish [35]. Considering the entire experimental period and all treatments, mean values (±standard deviation) were 4.64 ± 0.68 mg·L^−1^ for dissolved oxygen, 22.7 ± 0.6 °C for temperature, pH 7.05 ± 0.59, and electrical conductivity of 241.5 ± 80.9 µS·cm^−1^. All experimental procedures were approved by the Ethics Committee on the Use of Animals (CEUA) of UNESP/FCAV under protocol number 3195/2024.

### 2.2. Experimental Design

**Figure 1:**
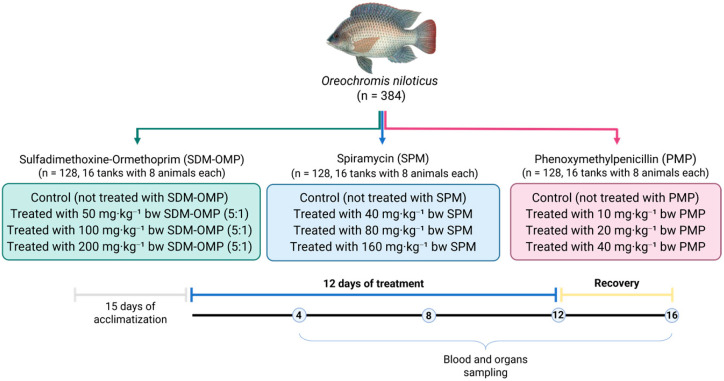
Schematic representation of the experimental design used to evaluate the clinical safety of sulfadimethoxine-ormetoprim (SDM-OMP), spiramycin (SPM), and phenoxymethylpenicillin (PMP) in Nile tilapia (*Oreochromis niloticus*). Fish were allocated into control and treated groups receiving different doses of each antibiotic (*n* = 128 fish per antimicrobial treatment, distributed in 16 tanks with 8 fish per tank). Treatments were administered for 12 days, with sampling performed on days 4, 8, and 12 of treatment (Highlighted in blue), followed by a 4-day recovery period (i.e., at 16 dati- days after treatment initiation, highlighted in yellow). At each sampling time point, eight fish per treatment were analyzed. Blood samples were collected for hematological and biochemical analyses, and spleen, liver, and kidney were collected for histopathological evaluation.

Prior to treatment, only clinically healthy fish without external signs of disease or abnormal behavior were randomly distributed among experimental tanks, which were subsequently assigned to control and treatment groups receiving different antimicrobial doses. For each antimicrobial, four experimental groups were established, consisting of one control group and three treated groups. Treatments were administered for 12 days, with sampling performed on days 4, 8, and 12 of treatment. Additionally, an extra group corresponding to the recovery period was sampled 4 days after completion of the 12-day treatment (i.e., at 16 dati- days after treatment initiation), according to the experimental design illustrated in Figure 1. At all experimental time points, blood samples were collected for hematological and biochemical analyses, and samples of spleen, liver, and kidney were collected for histopathological evaluation.

### 2.3. Definition of Experimental Doses

The doses of SDM–OMP were based on studies describing oral administration in Nile tilapia and other fish species using medicated feed at a total dose of approximately 50 mg·kg^−1^ body weight, administered at a fixed 5:1 ratio of sulfadimethoxine to ormetoprim (SDM:OMP) [14,36]. Doses of SPM were defined based on previous studies reporting its oral administration to tilapia via medicated feed at approximately 40 mg·kg^−1^ body weight [37]. For PMP, due to the scarcity of studies in fish, doses were defined based on pharmacological evidence and the use of the same molecule in other animal species, such as pigs, calves, dogs, and cats, with 10 mg·kg^−1^ adopted as the reference dose [38,39,40]. All doses were selected exclusively for the purpose of clinical safety evaluation.

### 2.4. Experimental Diet

A commercial extruded feed (Nutripiscis^®^; ADM, Descalvado, Brazil) containing 36% crude protein, 12% moisture, 60 g·kg^−1^ ether extract, 140 g·kg^−1^ ash, 50 g·kg^−1^ crude fiber, 10 g·kg^−1^ calcium, 8 g·kg^−1^ phosphorus, and 700 mg·kg^−1^ vitamin C was used to formulate the experimental diets for Nile tilapia. Feed was weighed daily according to the mean body weight of fish in each tank. The sulfadimethoxine–ormetoprim combination (SDM–OMP;) was incorporated into diets at 50, 100, and 200 mg·kg^−1^ body weight (5:1 ratio, SDM:OMP). Spiramycin (SPM;) was included at 40, 80, and 160 mg·kg^−1^ body weight, while phenoxymethylpenicillin potassium (PMP;) was added at 10, 20, and 40 mg·kg^−1^ body weight. Medicated diets were prepared using 1% carboxymethylcellulose (CMC, Labsynth, Diadema, Brazil) as a binding agent. According to the manufacturer, the CMC solution (10 g·L^−1^) was prepared in distilled water under continuous stirring until fully hydrated to thicken into a clear gel. The required amount of antimicrobial was first dispersed in the CMC gel solution and then mixed with a commercial basal feed to uniformly coat the pellets. The coated pellets were air-dried at room temperature before use. This procedure improves pellet adhesion and water stability, minimizing drug leaching prior to ingestion [41,42]. The control diet was prepared using the same procedure with 1% CMC but without antimicrobial addition to ensure comparable feed characteristics.

### 2.5. Fish Anesthesia

Tilapia were anesthetized by immersion in an aqueous benzocaine solution at a ratio of 1:10,000 for blood sampling and 1:500 for euthanasia. Benzocaine was previously dissolved in 98% ethanol (0.1 g·mL^−1^) and subsequently diluted in water, as described by Wedemeyer [43]. Blood sampling was performed after the loss of opercular reflexes, and euthanasia was conducted using a more concentrated anesthetic solution under aeration in order to minimize animal stress.

### 2.6. Blood Collection and Hematological Analysis

Eight fish per treatment, corresponding to one experimental tank, were anesthetized, and 1.5 mL blood samples were collected from the caudal vessel on days 4, 8, and 12 dati, as well as during the recovery period (day 16). Samples were divided into two aliquots: one collected in a syringe containing heparin (5000 IU) and the other without anticoagulant, for plasma and serum collection, respectively. For serum preparation, blood samples were allowed to clot at room temperature for 30–60 min. Erythrocyte counts (RBC) were performed using a Neubauer hemocytometer with Natt and Herrick’s solution [44] at a 1:100 (*v*/*v*) dilution. Hematocrit (HCT) was determined by the microcentrifugation technique, while circulating hemoglobin concentration (Hb) was measured spectrophotometrically at 540 nm (LabQuest^®^, Bioplus, Brazil) using a commercial reagent (Labtest Diagnóstica S.A., Lagoa Santa, MG, Brazil; Cat. No. 43). Mean corpuscular volume (MCV), mean corpuscular hemoglobin (MCH), and mean corpuscular hemoglobin concentration (MCHC) were calculated using the formulas MCV = (HCT/RBC) × 100, MCH = (Hb/RBC) × 10, and MCHC = (Hb/HCT) × 100, respectively. Differential leukocyte counts were performed on blood smears by counting 100 cells after staining with May–Grünwald–Giemsa–Wright [45]. All leukocyte differential counts were performed by a single trained and experienced laboratory technician to minimize operator-dependent variability.

### 2.7. Serum Biochemical Evaluation

Blood samples collected without an anticoagulant were centrifuged at 10,000 rpm for 10 min to obtain serum. Biochemical parameters, including alkaline phosphatase (ALP), aspartate aminotransferase (AST), creatinine, albumin, total protein, cholesterol, and triglycerides, were determined using commercial diagnostic kits (Labtest Diagnóstica S.A., Lagoa Santa, MG, Brazil; ALP—Cat. No. 79-4/30; AST—Cat. No. 109-2/100; creatinine—Cat. No. 96-300; albumin—Cat. No. 19-1/250; total protein—Cat. No. 99-250; cholesterol—Cat. No. 76-5/100; triglycerides—Cat. No. 87-5/100) according to the manufacturer’s instructions, using a semi-automatic biochemical analyzer (LabQuest^®^, Bioplus, Barueri, Brazil). Blood glucose levels were determined using a portable glucometer (Accu-Chek^®^ Performa, Roche Diagnostics, Mannheim, Germany).

### 2.8. Histopathological Evaluation of Organs

After 4, 8, and 12 dati and at the end of the recovery period (day 16), tilapia were euthanized by immersion in an aqueous benzocaine solution (1:500). Fish were subsequently dissected, and samples of liver, spleen, and posterior kidney were collected for histopathological evaluation. Tissue fragments were fixed in 10% buffered formalin for 24 h and then transferred to 70% ethanol until paraffin embedding. Histological sections approximately 5 µm thick were stained with hematoxylin and eosin (H&E). Histopathological evaluation was conducted qualitatively using light microscopy, focusing on the identification of clinically relevant morphological alterations indicative of tissue toxicity. No semiquantitative scoring system was applied.

### 2.9. Statistical Analyses

The experimental design for clinical safety evaluation was completely randomized. Each antimicrobial was considered an independent experiment for statistical analysis purposes. For each trial, a 4 × 4 factorial design was adopted, consisting of four treatments (control group and three doses of the respective antimicrobial) and four evaluation periods (4, 8, and 12 dati, and day 16 corresponding to the recovery period). Analysis of variance was performed using the GLM (General Linear Model) procedure of SAS software, version 9.3 (Statistical Analysis Software, 2012). When significant differences were detected (*p* < 0.05), means were compared using Tukey’s test, adopting a significance level of 5%.

## 3. Results

### 3.1. Clinical Safety of Sulfadimethoxine–Ormetoprim

#### 3.1.1. Hematological Analyses of Sulfadimethoxine–Ormetoprim

**Figure 2:**
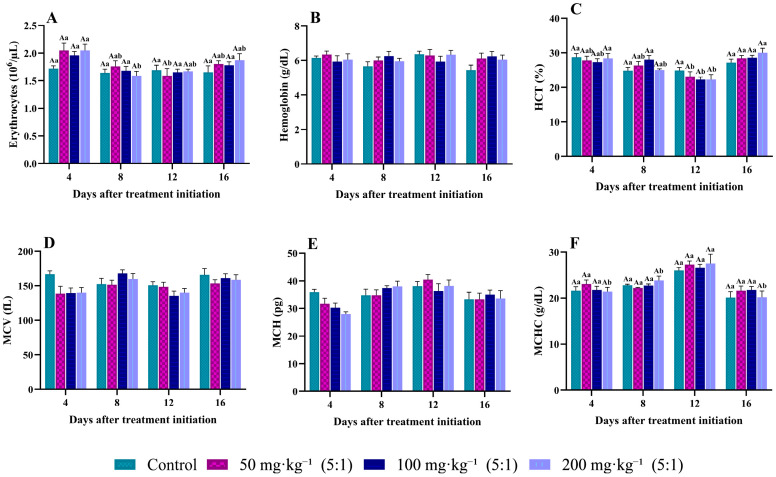
Erythrogram analysis of Nile tilapia treated with sulfadimethoxine–ormetoprim: (**A**) erythrocytes; (**B**) hemoglobin; (**C**) hematocrit (HCT); (**D**) mean corpuscular volume (MCV); (**E**) mean corpuscular hemoglobin (MCH); and (**F**) mean corpuscular hemoglobin concentration (MCHC). Data are presented as mean ± SE (*n* = 8). Means followed by the same letter do not differ significantly according to Tukey’s test (*p* < 0.05). Uppercase letters indicate comparisons among treatments within the same experimental day, whereas lowercase letters indicate comparisons over time within each treatment. In the graphs, bars without letters indicate that mean values did not differ significantly among treatments or over time.

Erythrocyte counts (Figure 2A) exhibited significant time-related variation. In fish treated with 50 mg·kg^−1^ (5:1, SDM:OMP), a progressive decrease was observed up to 12 dati (*p* = 0.0499), followed by partial recovery at 16 dati. Similarly, in the group treated with 200 mg·kg^−1^ (5:1, SDM:OMP), erythrocyte counts decreased at 8 dati, with a subsequent increase from 12 dati onward. Hematocrit values (Figure 2C) also showed significant temporal variation, characterized by a transient reduction at 12 dati in treated groups, followed by a return to baseline values at 16 dati. No significant changes were detected in hemoglobin concentrations throughout the experimental period (Figure 2B). Erythrocyte indices, represented by MCV (Figure 2D) and MCH (Figure 2E), did not differ significantly among treatments at any sampling time. In contrast, MCHC exhibited an isolated variation in the group treated with 200 mg·kg^−1^ (5:1, SDM:OMP), showing a significant increase at 12 dati.

#### 3.1.2. Leukogram Analyses of Sulfadimethoxine–Ormetoprim

**Figure 3:**
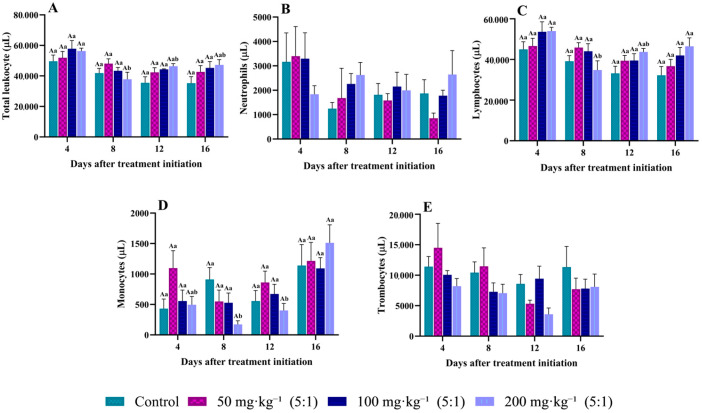
Leukocyte evaluation of tilapia treated with sulfadimethoxine–ormetoprim: (**A**) total leukocytes; (**B**) neutrophils; (**C**) lymphocytes; (**D**) monocytes; and (**E**) thrombocytes. Data are presented as mean ± SE (*n* = 8). Means followed by the same letter do not differ significantly according to Tukey’s test (*p* < 0.05). Uppercase letters indicate comparisons among treatments within the same experimental day, whereas lowercase letters indicate comparisons over time within each treatment. In the graphs, bars without letters indicate that mean values did not differ significantly among treatments or over time.

Leukocyte evaluation (Figure 3) revealed no significant differences among treatments on the same experimental day. However, a significant reduction in total leukocyte counts (Figure 3A) was observed in tilapia treated with 200 mg·kg^−1^ (5:1, SDM:OMP) at 8 dati (*p* = 0.0468) compared with other sampling times. This reduction was primarily associated with decreased lymphocyte (*p* = 0.0219) and monocyte (*p* = 0.0021) counts (Figure 3C,D). Lymphocyte counts increased at 16 dati, reaching values comparable to those observed at the beginning of the experimental period. Monocyte counts also showed a statistically significant increase at 16 dati compared with intermediate time points. No significant differences were detected in neutrophil or thrombocyte counts (Figure 3B,E) throughout the experimental period.

#### 3.1.3. Serum Biochemical Analyses of Sulfadimethoxine–Ormetoprim

**Figure 4:**
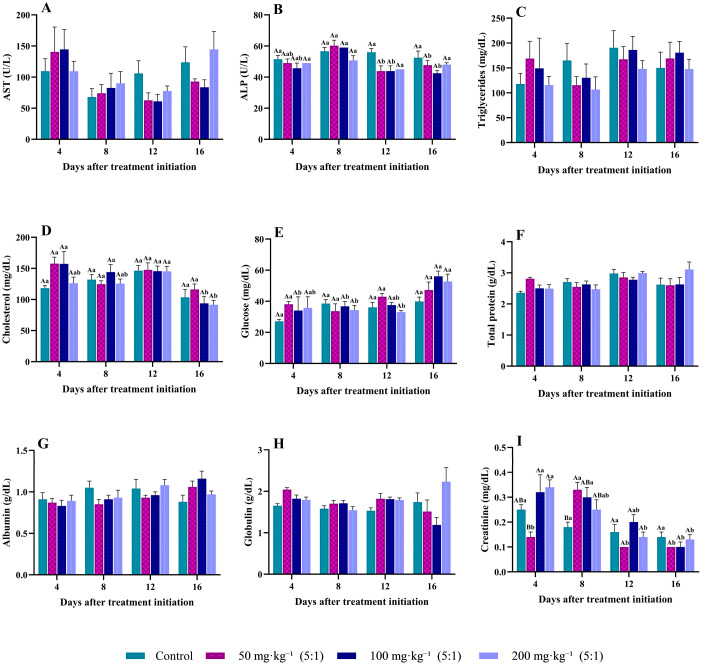
(**A**) aspartate aminotransferase (AST); (**B**) alkaline phosphatase (ALP); (**C**) triglycerides; (**D**) cholesterol; (**E**) glucose; (**F**) total protein; (**G**) albumin; (**H**) globulins; and (**I**) creatinine in tilapia treated with sulfadimethoxine–ormetoprim. Data are presented as mean ± SE (*n* = 8). Means followed by the same letter do not differ significantly according to Tukey’s test (*p* < 0.05). Uppercase letters indicate comparisons among treatments within the same experimental day, whereas lowercase letters indicate comparisons over time within each treatment. In the graphs, bars without letters indicate that mean values did not differ significantly among treatments or over time.

Analysis of hepatic enzyme activity in treated tilapia (Figure 4A,B) showed no significant changes in serum AST levels among treatments or over the experimental period. In contrast, ALP activity in SDM–OMP-treated tilapia exhibited significant temporal variation, although no differences were detected among treatments on the same experimental day. In the 50 mg·kg^−1^ and 100 mg·kg^−1^ (5:1, SDM:OMP) groups, a significant reduction in ALP activity was observed at 12 and 16 dati (*p* = 0.0367 and 0.0020; *p* = 0.0190 and 0.0030, respectively).

Serum triglyceride concentrations (Figure 4C) did not differ significantly among treatments or over the experimental period following SDM–OMP administration (*p* ≥ 0.05). Likewise, no significant differences in cholesterol or glucose levels were observed among treatments on the same experimental day (Figure 4D,E). However, significant temporal variation was detected in tilapia exposed to the highest SDM–OMP doses. In the 100 mg·kg^−1^ and 200 mg·kg^−1^ (5:1, SDM:OMP) groups, cholesterol levels (Figure 4D) decreased significantly at 16 dati (*p* = 0.0017 and *p* = 0.0492, respectively). In these same groups, glucose levels (Figure 4E) increased significantly at 16 dati (*p* = 0.0193 and *p* = 0.0365, respectively).

Plasma protein analysis (Figure 4F–H) revealed no significant differences in total protein, albumin, or globulin concentrations among treatments or over the experimental period (*p* > 0.05).

Serum creatinine levels varied both among treatments and over time (Figure 4I). At 4 dati, the 50 mg·kg^−1^ (5:1, SDM:OMP) group showed significantly lower values than the control group, whereas the 100 and 200 mg·kg^−1^ (5:1, SDM:OMP) groups did not differ from the control. At 8 dati, a reduction was observed in the control group, an increase in the 50 mg·kg^−1^ (5:1, SDM:OMP) group, and stable values in the 100 and 200 mg·kg^−1^ (5:1, SDM:OMP) groups. At 12 dati, creatinine levels decreased in the control group, as well as in the 50 and 200 mg·kg^−1^ (5:1, SDM:OMP) groups, with a slight reduction observed in the 100 mg·kg^−1^ (5:1, SDM:OMP) group. By 16 dati, creatinine concentrations stabilized in all groups, indicating recovery or physiological stabilization.

#### 3.1.4. Histopathological Analyses of Sulfadimethoxine–Ormetoprim

**Figure 5:**
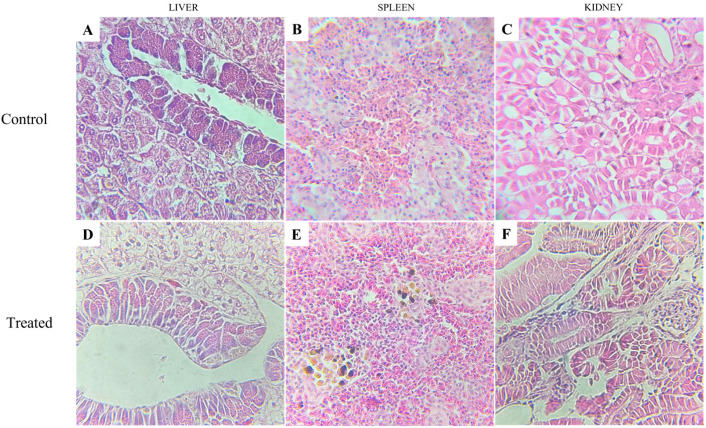
Representative photomicrographs of tilapia from the control group (**A**–**C**) and treated with sulfadimethoxine–ormetoprim (**D**–**F**): (**A**,**D**) liver; (**B**,**E**) spleen; and (**C**,**F**) kidney. Sections stained with hematoxylin and eosin (H&E), original magnification 40×.

Histopathological examination of SDM–OMP–treated tilapia (Figure 5) revealed no relevant morphological alterations in the evaluated organs.

### 3.2. Clinical Safety of Spiramycin

#### 3.2.1. Hematological Analyses of Spiramycin

**Figure 6:**
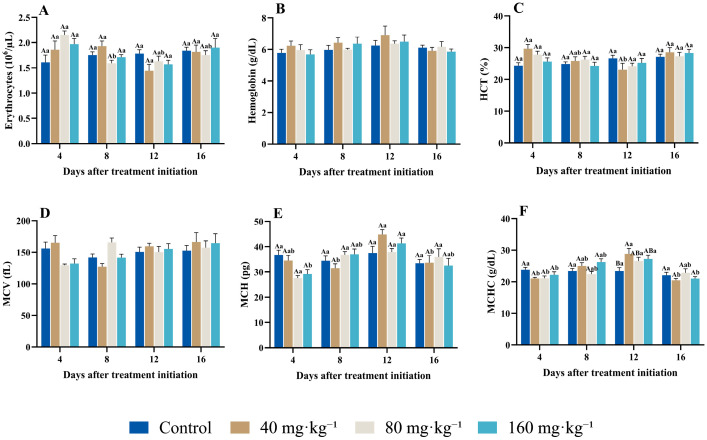
Erythrogram analysis of tilapia treated with spiramycin: (**A**) erythrocytes; (**B**) hemoglobin; (**C**) hematocrit (HCT); (**D**) mean corpuscular volume (MCV); (**E**) mean corpuscular hemoglobin (MCH); and (**F**) mean corpuscular hemoglobin concentration (MCHC). Data are presented as mean ± SE (*n* = 8). Means followed by the same letter do not differ significantly according to Tukey’s test (*p* < 0.05). Uppercase letters indicate comparisons among treatments on the same experimental day, whereas lowercase letters indicate comparisons over time within each treatment. In the graphs, bars without letters indicate that mean values did not differ significantly among treatments or over time.

Erythrocyte counts (Figure 6A) showed significant differences among the evaluated sampling times. In the group treated with 80 mg·kg^−1^, a reduction in erythrocyte counts was observed at 8 dati (*p* = 0.046), followed by stabilization at 12 and 16 dati. In the groups treated with 40 and 160 mg·kg^−1^, as well as in the control group, no significant differences were observed throughout the experimental period. Hematocrit values (Figure 6C) showed a significant reduction at 12 dati in the group treated with 40 mg·kg^−1^ (*p* = 0.0154), with a return to baseline values at 16 dati. In the groups treated with 80 and 160 mg·kg^−1^ and in the control group, hematocrit values remained stable over the experimental period. No significant changes were detected in hemoglobin concentrations (Figure 6B).

Erythrocyte indices represented by MCV (Figure 6D) did not differ among treatments throughout the experimental period. MCH (Figure 6E) differed among sampling times in the groups treated with 40 and 160 mg·kg^−1^. In the 40 mg·kg^−1^ group, an initial reduction was observed, followed by an increase at 12 dati and a subsequent decrease at 16 dati (*p* = 0.0246; *p* = 0.0036). In the group treated with 160 mg·kg^−1^, a significant increase in MCH was observed at 12 dati (*p* = 0.0055). Mean corpuscular hemoglobin concentration (MCHC; Figure 6F) showed significant differences among groups at 12 dati, with higher values in the group treated with 40 mg·kg^−1^ compared with the control group (*p* = 0.0281). In the groups treated with 40 and 160 mg·kg^−1^, MCHC increased at 12 dati and returned to baseline values at 16 dati. In the group treated with 80 mg·kg^−1^, MCHC increased at 12 dati, with intermediate values observed at 16 dati.

#### 3.2.2. Leukogram Analyses of Spiramycin

**Figure 7:**
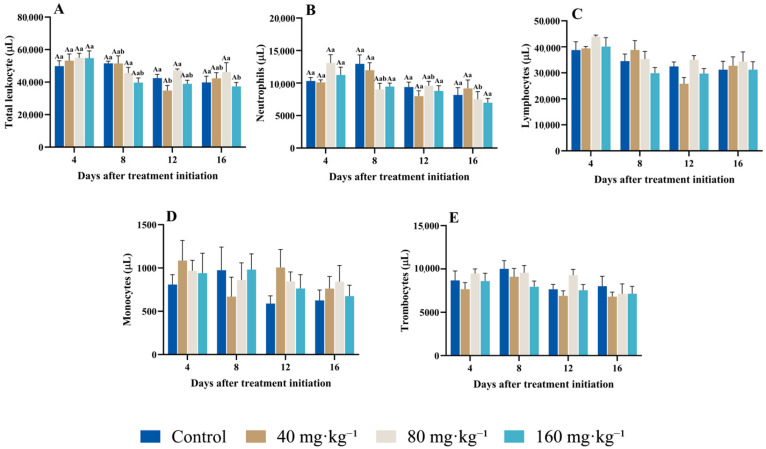
Leukocyte evaluation of tilapia treated with spiramycin: (**A**) total leukocytes; (**B**) neutrophils; (**C**) lymphocytes; (**D**) monocytes; and (**E**) thrombocytes. Data are presented as mean ± SE (*n* = 8). Means followed by the same letter do not differ significantly according to Tukey’s test (*p* < 0.05). Uppercase letters indicate comparisons among treatments on the same experimental day, whereas lowercase letters indicate comparisons over time within each treatment. In the graphs, bars without letters indicate that mean values did not differ significantly among treatments or over time.

Total leukocyte counts (Figure 7A) did not differ among treatments at the evaluated sampling times. In the group treated with 40 mg·kg^−1^, a significant reduction was observed at 12 dati (*p* = 0.0138). In the group treated with 160 mg·kg^−1^, a reduction in leukocyte counts was detected at 16 dati (*p* = 0.0176). No changes in total leukocyte counts were observed in the group treated with 80 mg·kg^−1^ or in the control group.

Neutrophil counts (Figure 7B) did not differ among treatments. However, in the group treated with 80 mg·kg^−1^, a significant reduction was observed, with lower values at 16 dati compared with initial sampling times (*p* = 0.026). No significant changes were observed in the groups treated with 40 and 160 mg·kg^−1^ or in the control group. Lymphocyte, monocyte, and thrombocyte counts (Figure 7C–E) showed no significant differences among treatments or sampling times.

#### 3.2.3. Serum Biochemical Analyses of Spiramycin

**Figure 8:**
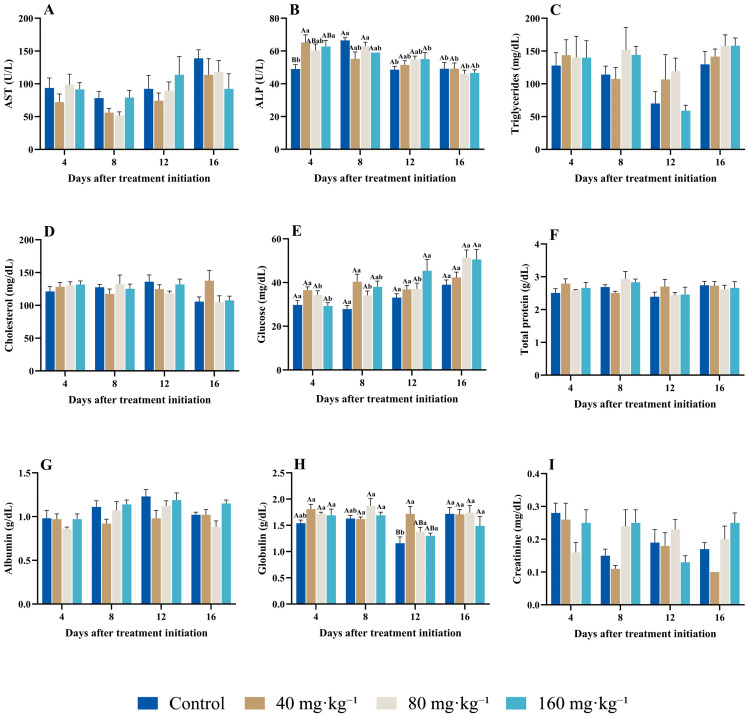
(**A**) aspartate aminotransferase (AST); (**B**) alkaline phosphatase (ALP); (**C**) triglycerides; (**D**) cholesterol; (**E**) glucose; (**F**) total protein; (**G**) albumin; (**H**) globulins; and (**I**) creatinine in tilapia treated with spiramycin. Data are presented as mean ± SE (*n* = 8). Means followed by the same letter do not differ significantly according to Tukey’s test (*p* < 0.05). Uppercase letters indicate comparisons among treatments within the same experimental day, whereas lowercase letters indicate comparisons over time within each treatment. In the graphs, bars without letters indicate that mean values did not differ significantly among treatments or over time.

AST activity (Figure 8A) showed no significant differences among treatments or among sampling times. ALP activity (Figure 8B) differed among treatments at 4 dati, with higher values observed in treated groups compared with the control group (*p* = 0.0264). At subsequent sampling times, no significant differences were observed among treatments. In all groups, ALP activity decreased relative to initial values, with the lowest levels observed at 16 dati and intermediate values at 12 dati.

Serum triglyceride and cholesterol concentrations (Figure 8C,D) showed no significant differences among treatments or among sampling times. Serum glucose levels (Figure 8E) did not differ among treatments at the evaluated sampling times. Over the course of the study, no changes were observed in the control group or in the group treated with 40 mg·kg^−1^. In the group treated with 80 mg·kg^−1^, glucose levels were lower at initial sampling times, followed by a significant increase at 16 dati (*p* = 0.0086; *p* = 0.0068). Similarly, in the group treated with 160 mg·kg^−1^, a progressive increase in glucose levels was observed throughout the experimental period (*p* < 0.0001; *p* = 0.0053).

Serum total protein and albumin concentrations (Figure 8F,G) showed no significant differences among treatments or throughout the experimental period. Serum globulin levels (Figure 8H) differed significantly among treatments at 12 dati, with lower values observed in the control group compared with the group treated with 40 mg·kg^−1^ (*p* = 0.0172). Over time, a reduction in globulin levels was observed in the control group (*p* = 0.0275), whereas values remained stable in the groups treated with 40, 80, and 160 mg·kg^−1^, with no significant changes.

Serum creatinine concentrations (Figure 8I) showed no significant differences among treatments or over the experimental period.

#### 3.2.4. Histopathological Analyses of Spiramycin

**Figure 9:**
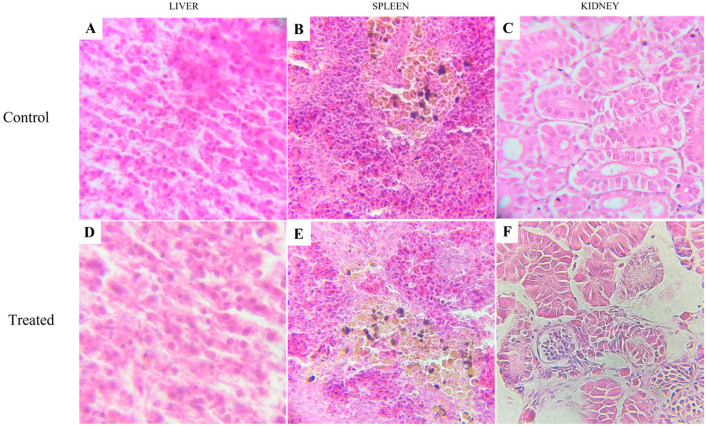
Representative photomicrographs of tilapia from the control group (**A**–**C**) and treated with spiramycin (**D**–**F**): (**A**,**D**) liver; (**B**,**E**) spleen; and (**C**,**F**) kidney. Sections stained with hematoxylin and eosin (H&E), original magnification 40×.

Histopathological examination of spiramycin-treated tilapia (Figure 9) revealed no relevant morphological alterations in the evaluated organs.

### 3.3. Clinical Safety of Phenoxymethylpenicillin Potassium

#### 3.3.1. Hematological Analyses of Phenoxymethylpenicillin Potassium

**Figure 10:**
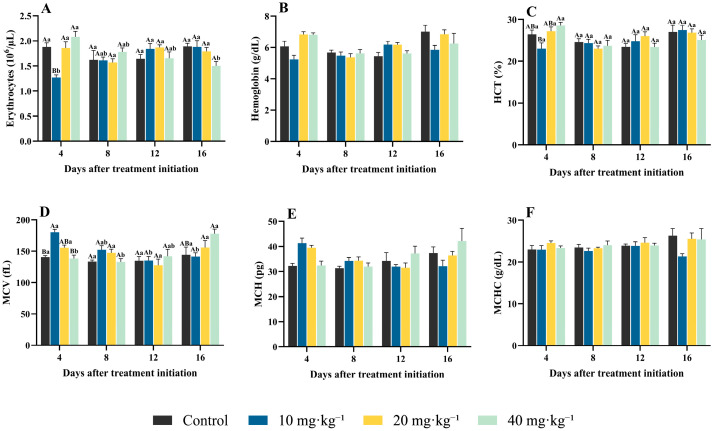
Erythrogram analysis of tilapia treated with phenoxymethylpenicillin potassium: (**A**) erythrocytes; (**B**) hemoglobin; (**C**) hematocrit (HCT); (**D**) mean corpuscular volume (MCV); (**E**) mean corpuscular hemoglobin (MCH); and (**F**) mean corpuscular hemoglobin concentration (MCHC). Data are presented as mean ± SE (*n* = 8). Means followed by the same letter do not differ significantly according to Tukey’s test (*p* < 0.05). Uppercase letters indicate comparisons among treatments on the same experimental day, whereas lowercase letters indicate comparisons over time within each treatment. In the graphs, bars without letters indicate that mean values did not differ significantly among treatments or over time.

Erythrocyte counts (Figure 10A) showed isolated differences among treatments over the experimental period. At 4 dati, a significant reduction in erythrocyte counts was observed in the group treated with 10 mg·kg^−1^ compared with the other groups (*p* < 0.0001; *p* = 0.0082; *p* = 0.0137). In the group treated with 40 mg·kg^−1^, a significant reduction in erythrocyte counts was detected at 16 dati (*p* = 0.0062). Hemoglobin concentrations (Figure 10B) did not show statistically significant differences among treatments or throughout the experimental period. Hematocrit values (Figure 10C) differed significantly among treatments only at 4 dati, with lower values observed in the group treated with 10 mg·kg^−1^ compared with the group treated with 40 mg·kg^−1^ (*p* = 0.0403). MCV (Figure 10D) showed significant differences among treatments at 4 dati, with lower values in the control group and in the group treated with 40 mg·kg^−1^ compared with the group treated with 10 mg·kg^−1^. Over the experimental period, the group treated with 10 mg·kg^−1^ exhibited a progressive reduction in MCV, with lower values observed at 12 and 16 dati. In contrast, the group treated with 40 mg·kg^−1^ showed a gradual increase in MCV throughout the study. MCH and MCHC (Figure 10E,F) did not differ significantly among treatments or among sampling times.

#### 3.3.2. Leukogram Analyses of Phenoxymethylpenicillin Potassium

**Figure 11:**
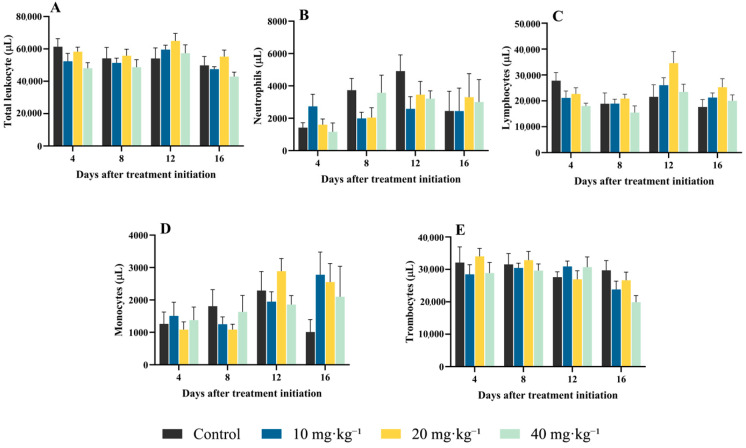
Leukocyte evaluation of tilapia treated with phenoxymethylpenicillin potassium: (**A**) total leukocytes; (**B**) neutrophils; (**C**) lymphocytes; (**D**) monocytes; and (**E**) thrombocytes. Data are presented as mean ± SE (*n* = 8). In the graphs, bars without letters indicate that mean values did not differ significantly according to Tukey’s test (*p* < 0.05) among treatments or over time.

Leukogram parameters (Figure 11A–E) showed no statistically significant differences among treatments or throughout the experimental period.

#### 3.3.3. Serum Biochemical Analyses of Phenoxymethylpenicillin Potassium

**Figure 12:**
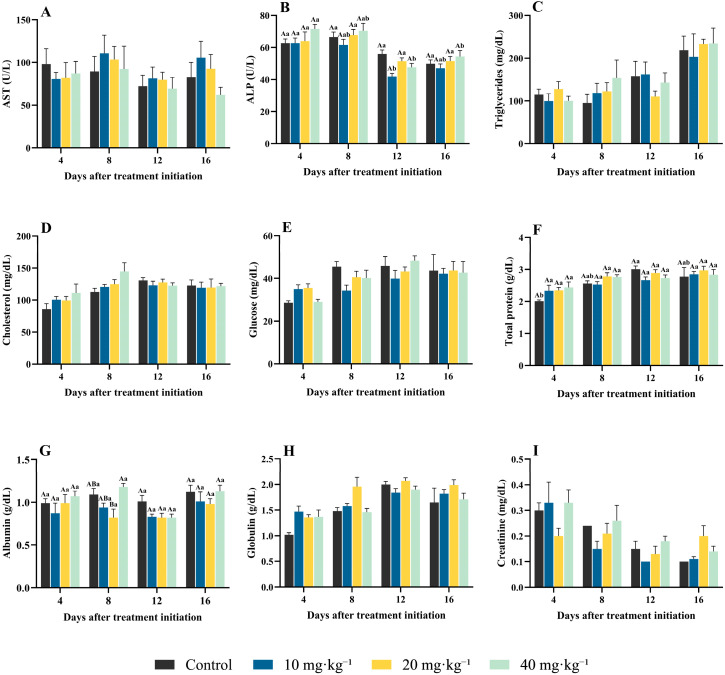
(**A**) aspartate aminotransferase (AST); (**B**) alkaline phosphatase (ALP); (**C**) triglycerides; (**D**) cholesterol; (**E**) glucose; (**F**) total protein; (**G**) albumin; (**H**) globulins; and (**I**) creatinine in tilapia treated with phenoxymethylpenicillin potassium. Data are presented as mean ± SE (*n* = 8). Means followed by the same letter do not differ significantly according to Tukey’s test (*p* < 0.05). Uppercase letters indicate comparisons among treatments within the same experimental day, whereas lowercase letters indicate comparisons over time within each treatment. In the graphs, bars without letters indicate that mean values did not differ significantly among treatments or over time.

AST activity (Figure 12A) remained stable, with no significant differences among treatments or over the experimental period. ALP activity (Figure 12B) also did not differ among treatments at the evaluated sampling times. Over the course of the study, the group treated with 10 mg·kg^−1^ showed a significant reduction in ALP activity at 12 dati (*p* = 0.0348), followed by partial recovery at 16 dati. Similarly, in the group treated with 40 mg·kg^−1^, ALP activity decreased at 12 and 16 dati (*p* = 0.0028; *p* = 0.032).

Serum triglyceride, cholesterol, and glucose concentrations (Figure 12C–E) did not show statistically significant differences among treatments or over the experimental period.

Serum total protein concentrations (Figure 12F) remained stable, with no significant differences among treatments at the evaluated sampling times. Over the experimental period, significant variation was observed only in the control group, characterized by an increase at 12 dati. Serum albumin concentrations (Figure 12G) showed a punctual difference among treatments at 8 dati, with lower values in the group treated with 20 mg·kg^−1^ compared with the group treated with 40 mg·kg^−1^ (*p* = 0.0335). Serum globulin concentrations (Figure 12H) did not show significant variation among treatments or throughout the experimental period.

Serum creatinine concentrations (Figure 12I) did not differ significantly among treatments or over the experimental period.

#### 3.3.4. Histopathological Analyses of Phenoxymethylpenicillin Potassium

**Figure 13:**
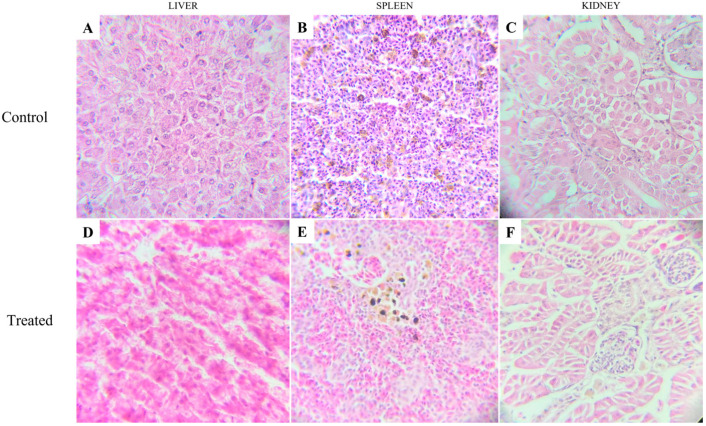
Representative photomicrographs of tilapia from the control group (**A**–**C**) and treated with phenoxymethylpenicillin potassium (**D**–**F**): (**A**,**D**) liver; (**B**,**E**) spleen; and (**C**,**F**) kidney. Sections stained with hematoxylin and eosin (H&E), original magnification 40×.

Histopathological examination of tilapia treated with phenoxymethylpenicillin potassium (Figure 13) revealed no relevant morphological alterations in the evaluated organs.

## 4. Discussion

The clinical safety assessment of antimicrobials in farmed fish is essential to support rational therapy while minimizing unintended impacts on welfare and production sustainability. In the present study, an integrated analysis (erythrogram, leukogram, serum biochemistry, and histopathology) allowed interpretation of the magnitude, time-course, and biological relevance of the detected changes, distinguishing transient responses of systemic toxicity.

Across antimicrobials, SDM–OMP produced the most consistent set of hematological and metabolic shifts, particularly at the higher doses. A transient leukopenia was detected in fish exposed to 200 mg·kg^−1^ (5:1, SDM:OMP) at 8 dati, driven mainly by reductions in lymphocytes and monocytes, followed by recovery toward baseline by 16 dati. This normalization of counts indicates a temporary leukocyte redistribution and/or immune adjustment. On the other hand, fish leukocyte parameters should be interpreted cautiously because they respond readily to husbandry and drug-related stress [30,46].

Biochemical responses in the SDM–OMP groups reinforced a “transient/adaptive” profile. AST remained stable, while ALP decreased over time in the 50 and 100 mg·kg^−1^ (5:1, SDM:OMP) groups (12–16 dati). In addition, cholesterol decreased and glucose increased at 16 dati in the 100 and 200 mg·kg^−1^ (5:1, SDM:OMP) groups. Together, these changes are most consistent with metabolic adjustments to treatment, rather than hepatic injury, particularly because histopathology of liver, spleen, and kidney showed no lesions in SDM–OMP–treated fish.

In the present study, aspartate aminotransferase (AST) showed greater variability among individuals compared with alkaline phosphatase (ALP). This difference may be explained by the distinct physiological and pathological processes. AST is a cytosolic and mitochondrial enzyme, and its plasma levels are highly sensitive to subtle physiological disturbances, including hepatocellular damage, metabolic stress, or minor tissue injury [47], which can contribute to greater inter-individual variability in experimental studies. In contrast, ALP is primarily associated with the biliary epithelium and is considered a marker of cholestasis or alterations in bile flow, rather than direct hepatocellular injury [48]. Because all antimicrobial treatments evaluated in this study did not appear to induce significant biliary dysfunction, ALP activity remained relatively stable across treatment groups. Previous studies suggest that small biochemical changes, especially with unchanged AST and normal histology, should be interpreted cautiously unless they are persistent and confirmed by tissue damage [30,31,46].

Sulfonamide effects reported in the literature vary by drug and treatment regimen. The largely reversible responses observed here resemble the mild hematological changes described for sulfamethazine under controlled exposure [27], whereas other sulfonamide combinations have produced more severe outcomes, including mortality, under different conditions [28].

SPM induced comparatively mild erythrogram changes with limited persistence. Erythrocyte counts decreased at 8 dati in the 80 mg·kg^−1^ group (with stabilization thereafter), and hematocrit decreased at 12 dati in the 40 mg·kg^−1^ group, returning to baseline by 16 dati; hemoglobin did not change significantly. Additional isolated variation in MCH and MCHC (notably at 12 dati) occurred without a consistent adverse pattern across hematimetric indices. Because the erythrocyte changes were mild, and were not accompanied by sustained changes in hemoglobin or hematimetric indices, they likely reflect a transient physiological response rather than clinical anemia or reduced oxygen-carrying capacity [30,31,46].

Leukogram results for SPM also indicated minor, time-limited changes: total leukocytes declined at 12 dati (40 mg·kg^−1^) and 16 dati (160 mg·kg^−1^), while neutrophils decreased over time in the 80 mg·kg^−1^ group. The absence of sustained lymphocyte/monocyte suppression, supports a non-progressive profile.

SPM mainly affected glucose at higher doses with increased level at 16 dati (80 mg·kg^−1^) and rose progressively in tilapia treated with 160 mg·kg^−1^, while triglycerides and cholesterol did not change.

Overall, these results suggest a stress response rather than liver cell damage, since AST was stable and ALP changes were transient (only an early increase at 4 dati) followed by decreases over time. Taken together, the SPM results support previous evidence that macrolides, when used under therapeutic regimens in fish, are typically well tolerated and associated predominantly with reversible physiological alterations [29,32,33].

Among evaluated drugs, PMP exhibited the most stable leukogram and biochemical profile. Leukocyte parameters showed no significant differences among treatments or across the experimental period.

Hematological effects were isolated and not progressive: erythrocyte count decreased at 4 dati in the 10 mg·kg^−1^ group and at 16 dati in the 40 mg·kg^−1^ group, while hemoglobin remained unchanged and hematocrit differed only at 4 dati. In the absence of a coordinated worsening of hemoglobin and erythrocyte indices, these variations do not support clinically significant impairment of oxygen transport and align with evidence that β-lactams are typically well tolerated in fish [30,32].

From a biochemical perspective, AST remained unchanged, and triglycerides, cholesterol, and glucose showed no differences among treatments or across time. The only consistent change was a decrease in ALP at later sampling points in treated fish. Because AST did not rise and histopathology revealed no relevant lesions, the ALP decrease is more consistent with adaptive metabolic modulation than with hepatotoxicity [30,31,46]. Renal safety was supported by stable creatinine and absence of renal histopathological alterations.

A central outcome of this work is the shared safety pattern observed across three distinct pharmacological classes evaluated under the same experimental framework. Despite drug-specific signals, all antimicrobials converged on a common profile: the changes, when present, were generally mild; effects were time-limited and tended to normalize by the recovery phase; and there was no evidence of organ injury, as biochemical stability of key markers was not accompanied by relevant hepatic or renal lesions on histopathology. Additionally, no mortality was observed throughout the experimental period across all treatment groups. From a practical standpoint, these comparative data support evidence-based selection when more than one therapeutic option is available: PMP appears to be associated with the lowest systemic disturbance under the tested regimen, SPM shows limited and largely reversible effects, and SDM–OMP warrants closer attention to immune/metabolic readouts at higher doses.

This study was conducted under controlled experimental conditions and over a defined exposure and recovery window, which strengthens internal comparisons among antimicrobials but limits direct extrapolation to field contexts. In commercial production, fish may experience repeated therapeutic cycles, variable water quality, fluctuating temperature/oxygen, and concurrent stressors that can amplify or reshape their physiological responses. Therefore, the cross-antimicrobial convergence observed here should be interpreted as evidence of clinical safety under the tested conditions, not as definitive proof of safety under all farming scenarios.

## 5. Conclusions

Under a 12-day oral administration followed by a 4-day recovery period, sulfadimethoxine–ormetoprim (SDM–OMP), spiramycin (SPM), and phenoxymethylpenicillin potassium (PMP) were clinically well tolerated in Nile tilapia, with no mortality or evidence of organ damage. Observed changes were mild, transient, and non-dose-dependent, with normalization during recovery and no corresponding histopathological alterations. Among treatments, PMP showed the most stable profile, SPM induced limited reversible responses, and SDM–OMP produced more detectable but still transient effects at higher doses. Overall, these findings support the clinical safety of the evaluated antimicrobials under controlled conditions and provide a base-line for their rational use in aquaculture.

## Data Availability

The data presented in this study are available from the corresponding author upon reasonable request.

## Abbreviations

The following abbreviations are used in this manuscript:

ALP	Alkaline phosphatase
AST	Aspartate aminotransferase
CEUA	Ethics Committee on Animal Use
CMC	Carboxymethylcellulose
dati	Days after treatment initiation
GLM	General Linear Model
H&E	Hematoxylin and eosin
Hb	Hemoglobin
HCT	Hematocrit
MCH	Mean corpuscular hemoglobin
MCHC	Mean corpuscular hemoglobin concentration
MCV	Mean corpuscular volume
PMP	Phenoxymethylpenicillin potassium
RBC	Red blood cell count
SDM-OMP	Sulfadimethoxine–ormetoprim
SPM	Spiramycin
